# The Association Between Family History of Upper Gastrointestinal Cancer and the Risk of Death from Upper Gastrointestinal Cancer–based on Linxian Dysplasia Nutrition Intervention Trial (NIT) Cohort

**DOI:** 10.3389/fonc.2022.897534

**Published:** 2022-06-08

**Authors:** Wan-yi Sun, Huan Yang, Xiao-kun Wang, Jin-hu Fan, You-lin Qiao, Philip R. Taylor

**Affiliations:** ^1^Department of Cancer Epidemiology, National Cancer Center/National Clinical Research Center for Cancer/Cancer Hospital, Chinese Academy of Medical Sciences and Peking Union Medical College, Beijing, China; ^2^Metabolic Epidemiology Branch, Division of Cancer Epidemiology & Genetics, National Cancer Institute, National Institutes of Health, Bethesda, MD, United States

**Keywords:** family history, lifestyle, upper gastrointestinal cancer, esophageal squamous dysplasia, Linxian Dysplasia Nutrition Intervention Trial

## Abstract

**Objective:**

Explore the influence of family history of upper gastrointestinal (UGI) cancer on UGI cancer death, based on the Linxian Dysplasia Nutrition Intervention Trial (NIT) cohort.

**Methods:**

Family history of UGI cancer was defined as at least one first-degree relative (parent, child, or sibling) had a history of esophageal or gastric cancer. Cancer death was carried out by ICD-10 code. Family history information was collected at baseline and cancer deaths were assessed at each annual follow-up. The COX proportional risk model was used to estimate the hazard ratio (HR) and 95% confidence interval (95% CI). We compared the positive family history group with the negative to determine the risk of family history on UGI cancer death. The effect of category of relatives, number of relatives with UGI cancer, and diagnosis age of relatives on the UGI death risk were further analyzed. Interaction and stratification analyses were done to see the subgroup effects. Sensitivity analyses were also conducted by exclusion of individuals who were followed up less than three years. We considered controlling of covariates including: gender, age (continuity), community, education level, number of siblings (continuity), BMI (continuity), smoking, alcohol use, fresh fruit intake, fresh vegetable intake, hot beverage intake, edible oil intake, meat intake, and moldy staple food intake. All food intake variables were converted into categorical variables.

**Results:**

From1985 to2015, we followed up total 3,318 individuals with 898 UGI cancer deaths (537 from ESCC, 77 from GNCC, and 284 from GCC). In a single factor analysis, family history of UGI cancer increased the risk of death of esophageal squamous cell carcinoma (ESCC) by 27% (HR=1.270, 95%CI1.072-1.504). No associations were observed in gastric cardia carcinoma (GCC) and gastric non-cardia carcinoma (GNCC). After adjusting for multi-factor, a family history of UGI cancer risk of death increased by 31.9% from ESCC (HR=1.319,95%CI:1.110-1.567). Subgroup analysis of different types of relatives with UGI cancers, UGI cancers in the mother (HR=1.457,95%CI:1.200-1.768), brother (HR=1.522,95%CI:1.117-2.073), and sister (HR=1.999,95%CI:1.419-2.817) were independent risk factors for ESCC death, while the father was not. In addition, 2 relatives with UGI cancer (HR=1.495, 95%, CI:1.110-2.013) and ≥3 relatives with UGI cancer (HR=2.836, 95%CI:1.842-4.367) significantly increased the risk of ESCC death, and the trend test was statistically significant (P<0.001). Relatives’ diagnostic age of 51-60 years (HR=1.322, 95%CI:1.046-1.672) and 41-50 years (HR=1.442, 95%CI:1.078-1.930) were the risk factors for ESCC death, with statistical significance in the trend test (P=0.010). No statistically significant result of the family history effect on the risk of death from GCC or GNCC was found. Sensitivity analysis of 80% of subjects, randomly selected, did not change the results.

**Conclusion:**

A family history of UGI cancer may predict the risk of death from ESCC but not from GCC or GNCC. UGI cancer in the mother may predict the risk of death from ESCC, but not father, which indicates gender differences. Gender and smoking are the interaction items with family history in a similar extent. In the subgroup, the risk of ESCC death is more distinct by family history in younger, female, and better-lifestyle individuals, which indicates the unique role of genetic factors.

## Introduction

Upper gastrointestinal cancer (UGI) includes gastric cancer and esophageal cancer with severe global cancer burden. Gastric cancer and esophageal cancer rank the second and sixth causes of cancer mortality in the world, respectively (1, 2). More than 73% of gastric cancer cases occur in Asia, while China accounts for half of the global cases. Gastric cancer includes gastric cardia carcinoma (GCC) and non-cardia carcinoma (GNCC), the former is the most prevalent in China (3). Esophageal cancer includes esophageal adenocarcinoma and esophageal squamous cell carcinoma (ESCC). Different from the dominant situation of esophageal adenocarcinoma in western countries (4), the vast majority of esophageal cancer cases in China are esophageal squamous cell carcinoma (5).

The distribution of morbidity and mortality of upper gastrointestinal cancers in China is also uneven, with a high incidence in central, eastern and central, and northern regions. Linxian, in Henan Province, is one of those areas, therefore, the death rate of ESCC is 10 times higher than the average death rate in China and 100 times higher than the death rate of white Americans (6). Meanwhile, the incidence of GCC and GNCC is also higher than other areas in China (7), with an annual incidence of more than 125/100,000 (8). Therefore, it is very important to understand the risk factors of the population of upper gastrointestinal cancer in high incidence areas in China for the prevention and disease control. In the 1980s, a study by the Chinese Academy of Medical Sciences (CAMS) and National Cancer Institute of USA(NCI) in Linxian, found that the population was deficient in vitamins and minerals and had a poor diet and lifestyle (8). In view of this previous research, CAMS and NCI established nutrition intervention trials based on both the general population and dysplasia population in Linxian.

At present, lifestyle, such as smoking, alcohol drinking, and adverse dietary factors (such as the lack of fresh fruit and vegetable intake) are common risk factors for upper gastrointestinal cancer (9–11). However, it is often difficult to distinguish between these risk factors alone. Therefore, exploring the comprehensive influence of environmental and genetic factors on UGI cancers and analyzing their interaction are very important for cancer prevention in high-risk areas. Family history not only represents the common genetic background of family members but also symbolize the common environment since family members live together (12, 13). The assessment of family history may indicate the role of both genetic and environmental factors in cancer, which is of great significance for the prevention and control of upper gastrointestinal cancer.

However, the extent to which family cancer history increases the risk of death from UGI cancer has not been determined. One reason is that previous studies looked at the role of genetics and environment in cancer separately and did not properly analyze the influence of family history and other lifestyle factors on UGI cancer. In Hebei province, China, a study found that younger onset age and multiple primary cancers of UGI cancers were associated with family history, which suggests the role of genetic factors (14). However, a study of a cohort of UGI cancer patients’; relatives in Taixing, Shandong Province, found that the family cohort of cancer patients with a low genetic risk score showed a higher cumulative risk than the family cohort with high genetic risk score, which suggests that environment and lifestyle play a major role in esophageal cancer rather than genetic factors (15). Therefore, while previous studies exist on heterogeneity, most studies did not collect lifestyle information in detail. Meanwhile, when considering family history of cancer, lifestyle was controlled as a confounding factor and the possible interaction between lifestyle and family history of cancer was ignored. Therefore, to analyze the degree family history of cancer increased the risk of UGI cancer death, we will need to collect comprehensive risk factors and use appropriate methods in the data analysis phase which carefully distinguishes confounding factors and interaction factors.

Esophageal squamous dysplasia is the precancerous lesion of ESCC (16). Previous studies have suggested that the esophageal squamous dysplasia population has significantly different basic demographic characteristics and lifestyle compared with the general population and the incidence and mortality of UGI cancer in the dysplasia group were higher. Through the comparison of the general group and dysplasia group in the Linxian Nutrition Intervention Trial, we found differences of age, body mass index, alcohol consumption, and fresh vegetable intake between the two groups (8, 17, 18). By comparing the incidence and death of cancers in these two groups, with 30 years of follow-up, it was found that esophageal cancer death in the general population accounted for 44.47% of the total death of malignant cancers and the death of gastric cancer accounted for 33.48%, while in high-risk groups, esophageal cancer and gastric cancer accounted for 51.2% and 34.0%, respectively (6, 19). At present, studies on the relationship between risk factors and UGI cancer in the general population have provided reliable and sufficient information (8, 20, 21). However, research on the relationship between risk factors and UGI cancer in the dysplasia population is limited. Some studies have suggested that body mass index, oral leukoplakia, and tooth loss increase the risk of death from UGI cancer in this population (6, 19, 22). However, no study suggests the association between family history of UGI cancer and the risk of its death. In view of the differences between the two groups and higher incidence and mortality of UGI cancer in the dysplasia group, more studies should uncover the association between risk factors and UGI cancer death.

This study intends to make use of the Linxian Dysplasia Nutrition Intervention Trial to analyze the influence of family history of UGI cancer on UGI cancer death.

## Materials and Methods

### Study Population

Detailed information about Linxian Dysplasia Nutrition Intervention Trial has been introduced in previous studies (20, 23). Briefly, a total of 3,318 subjects, aged 40 to 69 years, who were previously cytologically diagnosed with esophageal squamous dysplasia, were enrolled. The study excluded subjects who had been diagnosed with other debilitating diseases or regularly took any vitamins or minerals. A six-year randomized, double-blind, placebo-controlled, nutrition intervention trial (1985-1991) was conducted on all subjects. Participants either received placebos or 26 vitamins and minerals in doses typically two to three times the United States recommended daily allowances.

### Family History and Other Exposure Data at Baseline

Prior to intervention, subjects underwent a physical examination, questionnaire survey and included information such as demographic characteristics, socioeconomic status, health status, family history details, and lifestyle. Family history information contained the category of relatives (father, mother, sister, brother, spouse), diagnosis age, number of relatives with cancer, etc. A positive family history of UGI cancer was defined as at least one first-degree relative (parent, child, or sibling) had a history of esophageal or gastric cancer. Dietary information included intake of fresh fruits, fresh vegetables, hot beverages, edible oil, meat, and moldy staple food. In order to avoid bias caused by seasonal effects, this study calculated the consumption frequency during winter/spring and summer/autumn and converted the frequency into “times per year”. Smoking was defined as an individual who smoked or used a hookah or pipe weekly for at least six months. Alcohol drinking was defined as having consumed any alcoholic beverage in the past 12 months.

### Follow-Up

**Figure 1:**
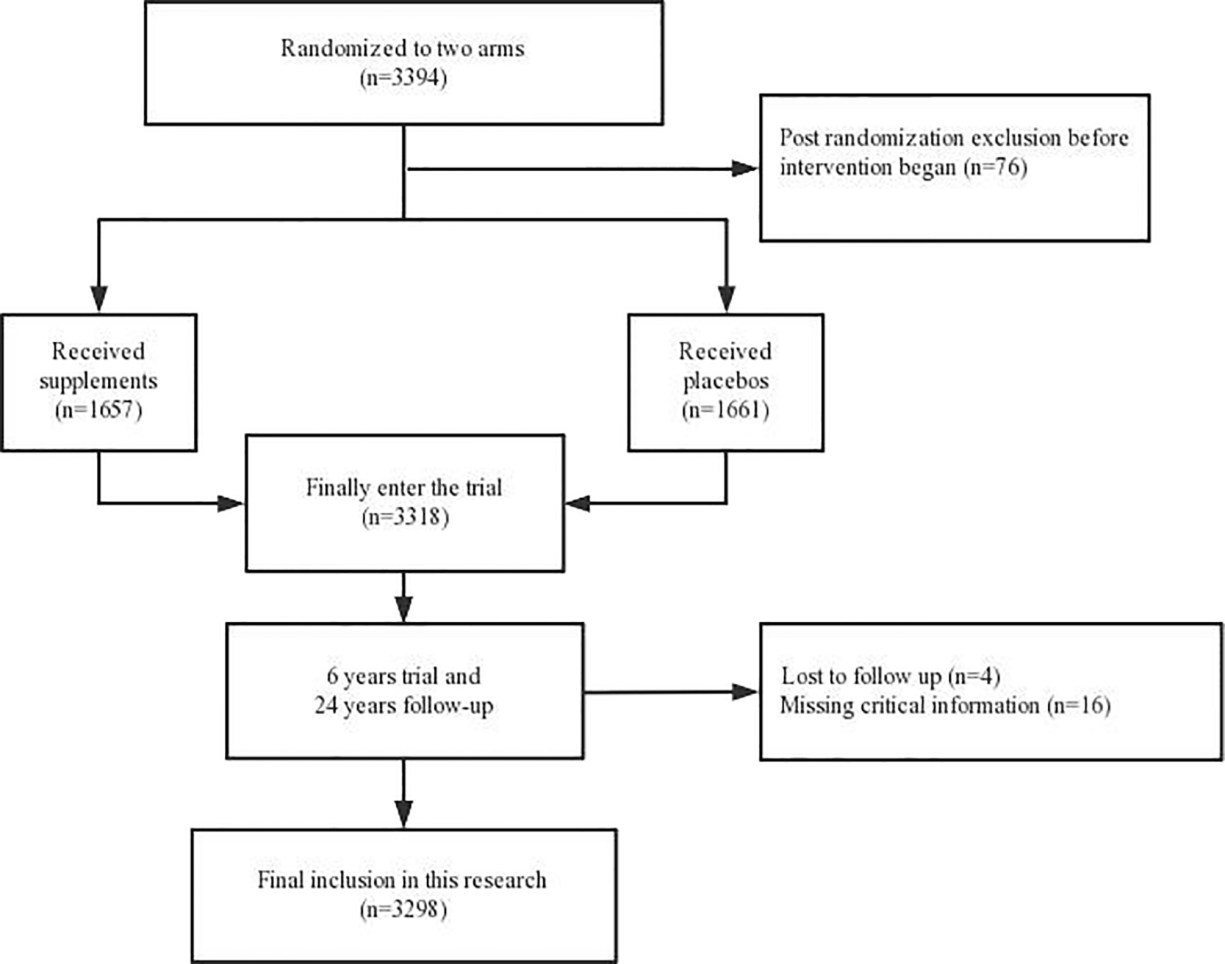
The flow diagram of the Linxian Dysplasia Nutrition Intervention Trial.

During the 6 year nutrition intervention trial period from 1985 to 1991, village health workers visited subjects monthly to determine life and disease status and all follow-up outcomes were confirmed by a panel of senior specialists in the United States and China. During the 24 years of follow-up (1991-2015), village health workers conducted the same steps and cancer diagnoses were verified by the same panel (1991-1996) or by senior Chinese diagnosticians from Beijing (1996-2015). Study outcome was the subject’s last known follow-up date, last follow-up date, date of death, or study end date, whichever occurred first. In our study, primary endpoints were ESCC, GNCC, and GCC deaths. All esophageal cancers were ESCC. In gastric cancer, cancers near the stomach from the cardia ≤3 cm were defined as GCC, while cancers in other parts of the stomach were defined as GNCC. All disease coding was carried out by ICD-10 code. **Figure 1** shows the flow diagram of the Linxian Dysplasia Nutrition Intervention Trial.

### Statistic Analysis

The t-test for continuous variables and chi-square test for classification variables were used to compare the characteristics of baseline demographic between positive and negative patients with family history of UGI cancer. The risk of death from UGI cancer was analyzed by the COX proportional risk regression model. According to UGI cancer/ESCC/GCC/GNCC, we analyzed the risk of death from different UGI cancers contributed by family history. We further distinguished family history information by category of relatives, diagnosis age, and the number of relatives with cancer to obtain more detailed results. Besides, interaction between family history and lifestyle items were analyzed and stratified by lifestyle items. Family history influences on the risk of death of UGI cancer in different level was found. Covariates included: gender, age (continuity), community, education level, number of siblings (continuity), BMI (continuity), smoking, alcohol drinking, fresh fruit intake, fresh vegetable intake, hot beverage intake, edible oil intake, meat intake and moldy staple food intake. All food intake variables were converted into categorical variables. Sensitivity analyses were also conducted by the exclusion of individuals who were followed up for less than three years. All results were analyzed by R X64 3.6.3 software. All tests were bilateral and P <0.05 was defined as statistically significant.

### Ethical Statement

The study was approved by the Institutional Review boards of the Cancer Hospital of the Chinese Academy of Medical Sciences and the National Cancer Institute (NCI) of the United States of America, and the written informed consent of all participants was obtained before their participation.

## Results

### Basic Demographic Characteristics and Lifestyle of the Studied Population

After the exclusion of 16 subjects with missing family history and lifestyle information at baseline, and 4 subjects with failed follow-up, a total of 3,298 subjects were included in the final analysis. Subjects were grouped according to positive/negative family history of UGI cancer, including 1,450 in the positive family history group and 1,848 in the negative family history group. The basic demographic characteristics and lifestyle of all subjects are shown in **Table 1**. Those with a positive family history had higher levels of education, more siblings, and all lifestyle differences except smoking and drinking, by contrast to negative family history group.

**Table 1:** Basic demographic characteristics and lifestyle of the studied population.

		Positive family history of UGI cancer (n=1450)	%	No family history of UGI cancer (n=1848)	%	*P* value
**Community**						0.118
	Yaocun	411	28.34	492	26.62	
	Runcun	541	37.31	657	35.55	
	Donggang	498	34.34	699	37.82	
**Gender**						0.849
	Male	633	43.66	814	44.05	
	Female	817	56.34	1034	55.95	
**Age**		52.87 ± 7.52		53.00 ± 7.45		0.614
**BMI**		20.37 ± 2.26		20.33 ± 2.32		0.635
**Education level**						0.036*
	Never	594	40.96	809	43.78	
	Not graduating from primary school	421	29.03	560	30.30	
	Primary school	137	9.45	131	7.09	
	Technical secondary school	81	5.59	80	4.33	
	Other	217	14.97	268	14.50	
**Number of siblings**						0.014*
	<2	208	14.34	333	18.02	
	2-4	942	64.97	1167	63.15	
	>4	300	20.69	348	18.83	
**Smoking**						0.392
	yes	431	29.72	523	28.30	
	no	1019	70.28	1325	55.14	
**Alcohol drinking**						0.275
	yes	283	19.52	332	17.96	
	no	1167	80.48	1516	82.03	
**Fresh fruit intake (times/year)**						0.003*
	0	414	28.55	595	32.20	
	1-6	535	36.90	712	38.53	
	7-12	201	13.86	197	10.66	
	>12	300	20.69	344	18.61	
**Fresh vegetable intake (times/year)**						0.002*
	0-Q1	69	4.76	100	5.41	
	Q1-Q2	376	25.93	539	29.17	
	Q2-Q3	320	22.07	436	23.59	
	>Q3	685	47.24	773	41.83	
**Hot beverage intake (times/year)**						0.010*
	0-Q1	482	33.24	663	35.88	
	Q1-Q2	473	32.62	632	34.20	
	Q2-Q3	236	16.28	285	15.42	
	>Q3	259	17.86	268	14.50	
**Edible oil intake (times/year)**						0.002*
	0	57	3.93	95	5.14	
	1-6	530	36.55	750	40.58	
	7-12	479	33.03	566	30.63	
	>12	384	26.48	437	23.65	
**Meat intake (times/year)**						0.012*
	0	152	10.48	185	10.01	
	1-6	632	43.59	877	47.46	
	7-12	483	33.31	648	35.06
	>12	183	12.62	138	7.47	
**Moldy staple food intake (times/year)**						0.032*
	0	1142	78.76	1509	81.66	
	1-6	132	9.10	155	8.39	
	7-12	108	7.45	114	6.17	
	>12	68	4.69	70	3.79	

P<0.05* was considered statistically significant. Q1\Q2\Q3 represent the first, second, third quartile respectively.

UGI, Upper gastrointestinal cancer; BMI, Body Mass Index.

### The Effect of Family History of UGI Cancer on the Risk of Death of UGI Cancer

There were 898 deaths from UGI cancers, including 537 from ESCC, 77 from GNCC, and 284 from GCC. The median follow-up time in the negative family history group was 23 years, 2 months, and 19 days and for positive group was 24 years, 10 months, and 5 days. **Table 2** shows the impact of those with positive family history on the risk of death in UGI cancers as a whole and in different types of cancers, compared to those with negative family history of UGI cancer. In ESCC, family history was a risk factor for its death, increasing by 27% (HR=1.270,95%CI:1.072-1.504) and 28.3% (HR=1.283,95%CI:1.083-1.519) in crude analysis and adjustment for sex and age and by 31.9% (HR=1.319,95%CI:1.110-1.567) in multi-factor adjustment. No statistically significant results were found in UGI cancers as a whole/GNCC/GCC.

**Table 2:** The effect of family history of UGI cancer on the risk of death of UGI cancer.

	Negative family history of UGI cancer [HR (95%CI)](n=1450)	Positive family history of UGI cancer [HR (95%CI)] (n=1848)	*P*
**UGI(n=898)**			
Single factor analysis	1.000	1.106 (0.970-1.261)	0.132
Adjustment for sex and age	1.000	1.114 (0.977-1.270)	0.106
Multi-factor adjustment **	1.000	1.128 (0.987-1.289)	0.077
**ESCC(n=537)**			
Single factor analysis	1.000	1.270 (1.072-1.504)	0.006*
Adjustment for sex and age	1.000	1.283 (1.083-1.519)	0.004*
Multi-factor adjustment **	1.000	1.319 (1.110-1.567)	0.002*
**GNCC(n=77)**			
Single factor analysis	1.000	1.236 (0.790-1.932)	0.353
Adjustment for sex and age	1.000	1.230 (0.787-1.923)	0.364
Multi-factor adjustment **	1.000	1.321 (0.834-2.093)	0.235
**GCC(n=284)**			
Single factor analysis	1.000	0.823 (0.648-1.045)	0.110
Adjustment for sex and age	1.000	0.819 (0.645-1.040)	0.102
Multi-factor adjustment **	1.000	0.797 (0.625-1.018)	0.069

P < 0.05* was considered statistically significant.

Multi-factor adjustment ** includes: gender, age(continuity), community, education level, number of siblings(continuity), BMI (continuity), smoking(classification), alcohol drinking(classification), fresh fruit intake, fresh vegetable intake, hot beverage intake, edible oil intake, meat intake and moldy staple food intake.

All food intake variables were converted into categorical ones.

UGI, Upper gastrointestinal cancer; ESCC, esophageal squamous cell carcinoma; GCC, gastric cardia carcinoma; GNCC, gastric non-cardia carcinoma; HR, hazard ratio; 95%CI, 95% confidence interval.

**Table 3** shows the effect of detailed family history information on the death risk of UGI cancers, including the effect of the category of relatives, number of relatives with UGI cancer, and diagnosis age of relatives on the death risk of ESCC/GNCC/GCC. Here, when there were multiple relatives with UGI cancer in one family, the item “diagnosis age of relatives” was taken at the earliest onset relative as the reference. The results were adjusted for many factors. In the ESCC category of relatives, mother (HR=1.457,95%CI:1.200-1.768), brother (HR=1.522,95%CI:1.117-2.073) and sister (HR=1.999,95%CI:1.419-2.817) were risk factors for ESCC death, while no statistically significant results were seen in fathers. Also, the number of relatives with UGI cancer was 2 (HR= 1.495,95%CI:1.110-2.013) and ≥3 (HR=2.836,95%CI:1.842-4.367) were risk factors that increased the risk of ESCC death and trend test was statistically significant (P<0.001). As for the diagnosis age of relatives, 51-60 years old (HR=1.322,95%CI:1.046-1.672) and 41-50 years old (HR=1.442,95%CI:1.078-1.930) were the risk factors for ESCC death and the trend test was statistically significant (P=0.010). In GCC, the positive family history with 1 relative of UGI cancer was a protective factor for GCC mortality compared with the negative family history group. No statistically significant results were found in GNCC.

**Table 3:** Effect of detailed items of family history on the risk of death from UGI cancer.

Items of family history	Number of death cases	HRs (95%CI)**
**ESCC(n=537)**		
**Category of relatives**		
Father	117	1.157 (0.938-1.428)
Mother	144	1.457 (1.200-1.768)
Brother	46	1.522 (1.117-2.073)
Sister	37	1.999 (1.419-2.817)
Sibling	74	1.688 (1.310-2.175)
Spouse	38	1.073 (0.765-1.505)
**Number of relatives with UGI cancer**		
0(Reference)	270	1.000
1	190	1.171 (0.969-1.413)
2	54	1.495 (1.110-2.013)
≥3	23	2.836 (1.842-4.367)
*p* for trend		<0.001*
**Diagnosis age of relatives**		
None (Reference)	279	1.000
>70	28	1.121 (0.757-1.661)
61-70	71	1.129 (0.866-1.471)
51-60	97	1.322 (1.046-1.672)
41-50	55	1.442 (1.078-1.930)
≤40	7	0.796 (0.374-1.694)
*p* for trend		0.010*
**GNCC(n=77)**		
**Category of relatives**		
Father	17	1.188 (0.681-2.072)
Mother	15	1.020 (0.573-1.816)
Brother	8	1.904 (0.887-4.088)
Sister	3	1.068 (0.330-3.455)
Sibling	11	1.770 (0.908-3.451)
Spouse	6	1.413 (0.596-3.351)
**Number of relatives with UGI cancer**		
0(Reference)	39	1.000
1	28	1.237 (0.755-2.024)
2	9	1.872 (0.894-3.921)
≥3	1	1.279 (0.174-9.429)
*p* for trend		0.155
**Diagnosis age of relatives**		
None (Reference)	39	1.000
>70	5	1.484 (0.577-3.813)
61-70	9	1.034 (0.498-2.148)
51-60	17	1.754 (0.982-3.132)
41-50	4	0.819 (0.292-2.298)
≤40	3	2.618 (0.792-8.650)
*p* for trend		0.213
**GCC(n=284)**		
**Category of relatives**		
Father	48	0.859 (0.625-1.181)
Mother	56	0.983 (0.732-1.320)
Brother	16	0.947 (0.567-1.580)
Sister	10	0.941 (0.498-1.781)
Sibling	25	0.970 (0.638-1.476)
Spouse	18	0.898 (0.551-1.464)
**Number of relatives with UGI cancer**		
0(Reference)	174	1.000
1	80	0.745 (0.569-0.975)
2	24	1.014 (0.656-1.566)
≥3	6	1.161 (0.508-2.655)
*p* for trend		0.430
**Diagnosis age of relatives**		
None (Reference)	176	1.000
>70	15	0.846 (0.497-1.440)
61-70	33	0.789 (0.542-1.147)
51-60	37	0.780 (0.545-1.116)
41-50	18	0.818 (0.503-1.331)
≤40	5	0.865 (0.353-2.122)
*p* for trend		0.092

P < 0.05* was considered statistically significant.

Multi-factor adjustment ** includes: gender, age(continuity), community, education level, number of siblings(continuity), BMI (continuity), smoking(classification), alcohol drinking(classification), fresh fruit intake, fresh vegetable intake, hot beverage intake, edible oil intake, meat intake and moldy staple food intake.

All food intake variables were converted into categorical ones.

UGI, Upper gastrointestinal cancer; ESCC, esophageal squamous cell carcinoma; GCC, gastric cardia carcinoma; GNCC, gastric non-cardia carcinoma; HR, hazard ratio; 95%CI, 95% confidence interval.

### Interaction Analysis and Stratified Analysis

**Table 4** shows the results of the interaction and stratified analysis. Since family history of UGI cancer only had major statistical significance with the risk of death of ESCC **Table 2** and **Table 3**), and the premise of interaction analysis was that both of the factors had an impact on the outcome, only the interaction between gender/age/lifestyle and family history of cancer in ESCC was analyzed. Therefore, stratification was performed according to gender/age/lifestyle to determine the impact of family history in different stratification on the risk of ESCC death.

**Table 4:** Interaction analysis and stratified analysis.

	Number of death	*P*	HRs(95%CI)**	Interaction *P* value
ESCC (n=537)				
**Age**			0.926 (0.655-1.310)	0.793
<53	229	0.013*	1.398 (1.072-1.824)	
≥53	308	0.062	1.246 (0.989-1.571)	
**Gender**			1.407 (1.207- 1.987)	0.024*
Male	254	0.730	1.046 (0.810-1.352)	
Female	283	< 0.001*	1.589 (1.249-2.021)	
**Smoking**			0.691 (0.476-0.997)	0.025*
no	372	< 0.001*	1.526 (1.239-1.878)	
yes	165	0.889	0.977 (0.710-1.346)	
**Alcohol drinking**			0.741 (0.468-1.173)	0.166
no	448	0.001*	1.395 (1.154-1.686)	
yes	89	0.856	1.041 (0.673-1.611)	

P < 0.05* was considered statistically significant.

Multi-factor adjustment ** includes: gender, age(continuity), community, education level, number of siblings(continuity), BMI (continuity), smoking(classification), alcohol drinking(classification), fresh fruit intake, fresh vegetable intake, hot beverage intake, edible oil intake, meat intake and moldy staple food intake.

All food intake variables were converted into categorical ones.

ESCC, esophageal squamous cell carcinoma; HR,hazard ratio; 95%CI, 95% confidence interval.

In the multiplicative interaction, family history of cancer was associated with smoking (P=0.025) and gender (P=0.024). In stratified analysis by item, subjects <53 years of age (HR=1.398,95%CI:1.072-1.824), female (HR=1.589,95%CI:1.249-2.021), non-smokers (HR=1.526,95%CI:1.239-1.878), and non-drinkers (HR = 1.395, 95% CI: 1.154 1.686) showed statistically significant results that family history of UGI cancers increased the risk of death from ESCC.

### Sensitivity Analysis

In order to ensure the stability of the results, we excluded individuals who were followed up for less than three years (n=153). There was no statistical difference between two groups **Table 5**.

**Table 5:** Results of sensitivity analysis.

	Number of death cases	HRs (95%CI)	HRs (95%CI)	HRs (95%CI)
		Univariate analysis	Adjust for gender and age	Multi-factor adjustment **
**UGI**				
Total	898	1.106 (0.970-1.261)	1.114 (0.977-1.270)	1.216 (1.033-1.432)*
Exclusion of subjects with less than 3-year follow up	823	1.100 (0.959-1.261)	1.108 (0.966-1.270)	1.120 (0.974-1.287)
**ESCC**				
Total	537	1.270 (1.072-1.504)*	1.283 (1.083-1.519)*	1.489 (1.212-1.830)*
Exclusion of subjects with less than 3-year follow up	500	1.246 (1.046-1.485)*	1.258 (1.055-1.499)*	1.289 (1.078-1.542)*
GNCC				
Total	77	1.236 (0.790-1.932)	1.230 (0.787-1.923)	1.326 (0.841-2.091)
Exclusion of subjects with less than 3-year follow up	71	1.038 (0.650-1.656)	1.033 (0.647-1.649)	1.110 (0.686-1.796)
**GCC**				
Total	284	0.823 (0.648-1.045)	0.819 (0.645-1.040)	0.806 (0.632-1.027)
Exclusion of subjects with less than 3-year follow up	252	0.868 (0.674-1.116)	0.863 (0.671-1.111)	0.840 (0.649-1.087)

P<0.05* was considered statistically significant.

Multi-factor adjustment ** includes: gender, age(continuity), community, education level, number of siblings(continuity), BMI (continuity), smoking(classification), alcohol drinking(classification), fresh fruit intake, fresh vegetable intake, hot beverage intake, edible oil intake, meat intake and moldy staple food intake.

All food intake variables were converted into categorical ones.

UGI, Upper gastrointestinal cancer; ESCC, esophageal squamous cell carcinoma; GCC,gastric cardia carcinoma; GNCC, gastric non-cardia carcinoma; HR,hazard ratio; 95%CI, 95% confidence interval.

## Discussion

By comparing the eating habits, lifestyle, and basic demographic characteristics of the patients with a positive family history and those with a negative family history, this study found that the characteristics of the patients with a positive family history were different from those with a negative family history but not all the characteristics were more adverse to health. People with a positive family history had higher levels of education, higher intake of fresh fruit and vegetables, lower intake of meat (even though they also had more siblings), higher intake of hot drinks, edible oil, and moldy staple food intake, which we often regard as risk factors. This is similar to the studies in Jiangsu, China and Kashmir, India (12, 24), which further indicates that the effect of family history is not only caused by genetic factors but also that the family with UGI cancer history had similar family lifestyles, some of which were not conducive to the prevention and treatment of upper gastrointestinal cancers.

For the analysis of the risk of death from UGI cancer, it can be seen in ESCC that, whether crude analysis or adjusted for gender, age, or adjusted for multiple factors, a positive family history increases the risk of UGI cancer death compared with that of the negative group, which is consistent with the research results of Shanxi, China, another high-risk area (25). In the analysis of family history and death risk of GNCC, there was no statistically significant result in this study, which can be explained by a case-control study in the United States (26). This study suggested that the risk of death of GNCC was associated with family history of gastric cancer but not with family history of esophageal cancer. In this study, the main type of UGI cancer in the positive family history group was ESCC (90.2%), while none of the relatives with positive family history had GNCC. Therefore, the increased risk of GNCC death was not found in this study, which was related to the fact that the main type of family history cancer was not GNCC. The same negative results were also found in GCC. However, the risk of GCC death was confirmed to be related to a family history of ESCC because the cardia is anatomically close to the esophagus and is considered to have the same risk factors (27). Therefore, the interpretation of this study is as follows: 1) some important risk factors of GCC were not fully included, such as H. pylori infection (28), which may confuse the true association between family history and the risk of death; 2) the study population had its particularity and since few studies are based on the population with severe esophageal squamous cell hyperplasia, it cannot be ruled out that the population has such characteristics; and 3) previous studies have suggested that the contribution of UGI cancer family history to the pathogenesis of UGI cancer decreases from ESCC to GCC (29). Therefore, compared with ESCC, the association between family history and GCC death risk is more likely to reach the conclusion that H0 is not rejected, especially when the sample size is limited.

Few domestic studies have carried out detailed analysis of family history to provide their effect on the risk of death of UGI cancer, such as the category of relatives, diagnosis age of relatives, and number of cancer patients. According to the literature retrieved in this study, there are three relevant pieces literature (12, 15, 30) which are divided into parents and siblings in the analysis of the relatives category, without further distinguishing the gender of relatives. In this study, regarding the family history of ESCC, we separated out relatives according to gender. In the positive family history group, when there was a mother (HR=1.457,95%CI:1.200-1.768), brother (HR=1.522,95%CI:1.117-2.073) or sister (HR=1.999,95%CI:1.419-2.817) with UGI cancer, there was an increased risk of death of ESCC, while there was no statistically significant outcome in fathers or spouses. The previous findings suggest that a father with UGI cancer does not increase the risk of subjects’ ESCC death, while mother does. The comparison between brothers and sisters was not statistically significant, so the impact of them is similar. At present, there are no direct studies based on esophageal squamous dysplasia population to provide an explanation. However, a study on key genes for the occurrence of ESCC indicated that the occurrence of ESCC was related to the specific methylation of heterochromatin binding marker regions (H3K9me3, H3K27me3), and the methylation marker regions were inherited from the mother (31), which might partly explain our results regarding the number of cancer patients. The group with 2 relatives with UGI cancer (HR=1.495,95%CI:1.110-2.013) or even ≥3(HR=2.836,95%CI:1.842-4.367), had higher ESCC death risk, compared with those with a negative family history and the trend test was statistically significant. As for the analysis of diagnosis age of relatives, this study also found that the younger the relative’s age at diagnosis, the greater the risk of ESCC death of the subject. The results of diagnosis age and number of relatives are consistent with the results of previous studies (12).

The multiplicative interaction showed that gender and smoking had an interaction with family history, suggesting that the relationship between family history and these items should be fully considered in future studies of ESCC. From the effect value of gender (HR=1.407,95%CI: 1.207-1.987) and smoking (HR=0.691,95%CI=0.476-0.997), we can conclude that the interaction of gender and smoking with family history has similar extent. Therefore, further stratified analyses needs to be performed to see the effect of family history on the risk of death from cancer within different layers, rather than merely controlling for all these items roughly as confounding factors. In addition, the stratified analysis concluded that family history increased the risk of ESCC death among people < 53, female, non-smoking, and non-drinking. Therefore, this study also suggests that the prominent influence of family history on the risk of death from ESCC should be noted in young, female, and related lifestyle groups.

Based on the stratified analysis results, the role of family history genetic effects in ESCC was further demonstrated. Due to the lack of lifestyle information of the subject’s relatives, this study could not distinguish the influence of genetic susceptibility and environmental exposure shared by family members. But in stratified analyses, a consistent risk of ESCC death from family history was observed among individuals not exposed to lifestyle risk factors, such as non-smoking (HR = 1.526, 95% CI: 1.239 1.878) and non-drinking (HR = 1.395, 95% CI: 1.154 1.686). The logical answer is that in the better lifestyle group, genetic role represented by family history looms large, so that they are at higher risk than those without a family history. In addition, family history raising ESCC death risk was also found in subjects of younger age (<53 years of age) (HR=1.398,95%CI:1.072-1.824). The onset of early cancer is linked to genetic factors rather than environmental exposures, highlighting family history as an indicator of genetic susceptibility, especially in the better lifestyle and younger onset age groups. It is also noteworthy that in gender stratification, family history increases the risk of ESCC death in women (HR=1.589,95%CI:1.249-2.021), and not in men, again suggesting the influence of gender on family history and UGI cancer death.

In summary, this study has the following highlights: First, it is a pioneer study as in the past, few have explored the relationship between family history and the risk of death from UGI cancer in the high-risk population of ESCC. Second, it shows a more detailed analysis and result than previous studies regarding family history items which include category of relatives, number of relatives with UGI cancer, and diagnosis age of relatives. Third, it discusses the relationship between family history and other lifestyle items/factors that may affect family history and death of UGI cancer. These were comprehensively analyzed and were not only controlled as confounding factors but were also analyzed in the interaction effect between family history and lifestyle and were further stratified to see the impact of family history on UGI cancer death in different lifestyle layers.

However, there are some limitations in this study: First, estimation of sample size. Few previous studies have explored the impact of family history on risk of death from UGI cancer in esophageal squamous dysplasia population. Therefore, we have no reliable studies to calculate sample size. Second, death caused by other diseases would have a competitive effect on the outcome of this study and the impact of the competitive effect on the study needs to be evaluated.

In a word, the family history of UGI cancer increased the risk of death from ESCC, but not from GCC or GNCC. UGI cancer in the mother increased the risk of death from ESCC but no effect from the father was observed. Further studies are needed to explore the mechanisms associated with family history of UGI cancer and the risk of UGI cancer death.

## Data Availability Statement

The original contributions presented in the study are included in the article/supplementary material, further inquiries can be directed to the corresponding authors.

## Ethics Statement

The studies involving human participants were reviewed and approved by the Institutional Review Boards of Cancer Hospital, Chinese Academy of Medical Sciences. The patients/participants provided their written informed consent to participate in this study.

## Author Contributions

W-YS was responsible for the design, data analysis and writing of the paper. X-KW and HY assisted W-YS in her work, including database building and data analysis. Professor J-HF provided guidance on the cohort study for the paper. Professor Y-LQ and PT reviewed the paper and provided revisions as well. All authors contributed to the article and approved the submitted version.

## Funding

This study was supported by a US National Cancer Institute contract (HHSN261201700047C) to the Cancer Hospital, Chinese Academy of Medical Sciences, China.

## Conflict of Interest

The authors declare that the research was conducted in the absence of any commercial or financial relationships that could be construed as a potential conflict of interest.

## Publisher’s Note

All claims expressed in this article are solely those of the authors and do not necessarily represent those of their affiliated organizations, or those of the publisher, the editors and the reviewers. Any product that may be evaluated in this article, or claim that may be made by its manufacturer, is not guaranteed or endorsed by the publisher.
